# Complete mitochondrial genomes of the Laotian Rock Rat (*Laonastes aenigmamus*) confirm deep divergence within the species

**DOI:** 10.1080/23802359.2016.1186520

**Published:** 2016-11-22

**Authors:** Minh Le, Fernando Penaloza, Renata Martins, Thanh V. Nguyen, Ha M. Nguyen, Dang X. Nguyen, Luong D. Nguyen, Andreas Wilting

**Affiliations:** a Department of Environmental Ecology, Faculty of Environmental Science, Hanoi University of Science, VNU, Hanoi, Vietnam;; b Centre for Natural Resources and Environmental Studies, VNU, Hanoi, Vietnam;; c Leibniz Institute for Zoo and Wildlife Research, Berlin, Germany;; d U.S. Agency for International Development, Governance for Inclusive Growth Program, Chemonics International Inc, Hanoi, Vietnam;; e Institute of Ecology and Biological Resources, Vietnam Academy of Science and Technology, Hanoi, Vietnam;; f Fauna and Flora International, Hanoi, Vietnam

**Keywords:** Diatomyidae, laotian rock rat, next-generation sequencing, rodentia, southeast Asia

## Abstract

Mitochondrial genomes of five Laotian Rock Rat (*Laonastes aenigmamus*) samples from Vietnam and Laos were sequenced using an Illumina platform. After *de novo* assembly, 13 protein-coding genes and two rRNA (12S and 16S) of the five genomes were aligned and analyzed with those from other related species under maximum likelihood and Bayesian inferences. Both methods revealed congruent tree topologies, which support two independently evolving clades of *L. aenigmamus* from Laos and Vietnam. The relaxed time calibration analysis showed that the two major lineages of the Laotian Rock Rat split about 8 million years ago, which was consistent with the results from previous studies using only cytochrome b sequences. Such a deep divergence time suggests the recognition of two rock rat species, but further nuclear DNA and morphological data are needed to solve the taxonomy of this taxon.

## Introduction

The Laotian Rock Rat (*Laonastes aenigmamus*), an enigmatic small mammal endemic to the Annamite Range, is a monotypic species of the family Diatomyidae, whose other members all went extinct about 11 MYA (Jenkins et al. 2005; Dawson et al. 2006; Nicolas et al. 2012). Early studies suggest that the species was restricted to the Khammuane Limestone area in central Laos (Jenkins et al. 2005; Rivière-Dobigny et al. 2011; Nicolas et al. 2012). However, recent surveys in Vietnam’s Phong Nha-Ke Bang National Park on the eastern side of the Annamites discovered a new population (Nguyen et al. 2012; Le et al. 2015). The discovery shows that this species might have a broader distribution in the poorly studied mountain range.

Previous genetic studies revealed multiple, distinct evolutionary lineages within the species, and indicate an ancient isolation, from *ca*. 8 to 12 MYA, between major clades from Laos and Vietnam (Nicolas et al. 2012; Le et al. 2015). The long temporal separation within a small distribution range (200 × 100 km) is especially interesting and likely a result of its ecological specialization to limestone environments and the fragmentation of these karst formations during the Late Miocene (Nicolas et al. 2012). However, previous studies only use one mitochondrial gene (cytochrome b) to calibrate divergence times, which can result in biased estimates of the temporal radiation (Marshall et al. 2016). In this study, we sequenced five complete mitochondrial genomes of the two major clades of the Laotian Rock Rat with samples from Lao PDR and Vietnam. We analyzed our newly generated data with multiple outgroups to determine the time divergence between the two lineages.

## Material and methods

### DNA extraction and NGS sequencing

**Figure 1.**
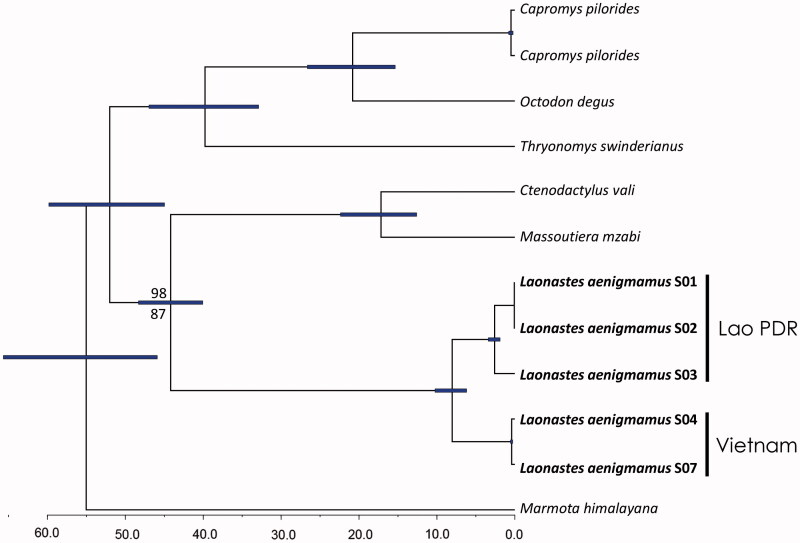
Time-calibrated chronogram using the constant size coalescent tree prior in BEAST. Bayesian and maximum-likelihood analyses showed an identical topology. Imperfect statistical support values are shown with numbers above and below branches representing those derived from Bayesian and maximum likelihood analyses, respectively. Numbers under the bottom axis represent million years before present. Samples on the tree have following corresponding vouchers: S01 – CDL1, S02 – CDL2, S03 – CDL3, S04 – CD1, S07 – CD21.

Five samples of *Laonastes aenigmamus*, two from Phong Nha-Ke Bang National Park, central Vietnam, and three from Hin Nam No National Biodiversity Conservation Area, central Laos were included in the study. The specimens were accessioned in the vertebrate collection of the Institute of Ecology and Biological Resources (IEBR) at the Vietnam Academy of Sciences and Technology (Figure 1). Information of their localities is provided in Le et al. (2015). For phylogenetic and time calibration analyses, we selected six species as outgroups based on their phylogenetic relationships established by Huchon et al. (2007). Sequences of outgroup taxa were downloaded from GenBank.

All tissue samples were extracted using the DNeasy Blood and Tissue kit (Qiagen, Hilden, Germany) following the manufacturer’s instructions with an overnight lysis and a 15 minute incubation period at 37 °C during the elution. Before sequencing library preparation, all samples were checked on a TapeSation (Agilent Technologies, Waldbronn, Germany) for fragment size distribution. Samples with large fragments were subsequently sheared with a M220 Focused-ultrasonicator (Covaris Ltd, Brighton, UK) to a peak target size of 300 bp, and re-checked for size distribution. Double-stranded Illumina sequencing libraries were prepared according to the protocol designed by Fortes and Paijmans (2015) with single 8 nt indices. All libraries were pooled equimolarly and sequenced on a MiSeq instrument (Illunina GmbH, Munich, Germany) using the v2 300-cycle kit.

### Mitogenome assembly and annotation

To de-multiplex and convert BCL files generated by the Illumina sequencer to FASTQ files bcl2fastq v2.17.1.14 (Illumina) was used. Adapter sequences present in the FASTQ files were removed using cutadapt v1.3) (Martin 2011). Low-quality stretches were removed, and reads shorter than 30 bp were discarded using PRINSEQ (Schmieder & Edwards 2011). The final paired-end reads of 150 bp were used as the input for mitoMaker, which performs a de-novo and reference-based assembly using SOAPdenovoTrans v1.03 (Xie et al. 2011) and MITObim v1.7 (Hahn et al. 2013). The assemblies were automatically annotated using tRNAscan-SE v1.4 (Lowe & Eddy 1997) and BLAST + v2.2.29 + (Camacho et al. 2009) using the metazoan mitochondrial genomes found in the NCBI RefSeq 39 as reference. The results from the automatic annotation were manually curated. The resulting five mitochondrial genomes have been cataloged in GenBank under accession numbers KU940253 - KU940257 of samples 1, 2, 3, 4, and 7, respectively (Figure 1). The details of the new genomes are shown in Table 1.

**Table 1.** Characterization of the Laotian Rock Rat’s mitochondrial genome.

		Position			Codon		
Gene	Length (bp)	Start	End	Intergenic Nucleotides	Start	Stop	Strand
tRNA-Phe	69	1	69	0			+
rRNA-12S	955	70	1024	0			+
tRNA-Val	74	1025	1098	0			+
rRNA-16S	1584	1099	2682	0			+
tRNA-Leu	75	2683	2757	0			+
CDS-ND1	960	2761	3720	3	ATG	TAA	+
tRNA-Ile	69	3720	3788	−1			+
tRNA-Gln	72	3857	3786	−3			−
tRNA-Met	69	3865	3933	7			+
CDS-ND2	1038	3934	4971	0	ATC	TAG	+
tRNA-Trp	68	4970	5037	−2			+
tRNA-Ala	69	5110	5042	4			−
tRNA-Asn	73	5183	5111	0			−
tRNA-Cys	67	5284	5218	34			−
tRNA-Tyr	66	5349	5284	−1			−
CDS-COI	1545	5351	6895	1	ATG	TAA	+
tRNA-Ser	69	6961	6893	−3			−
tRNA-Asp	68	6965	7032	3			+
CDS-COII	684	7033	7716	0	ATG	TAA	+
tRNA-Lys	66	7719	7784	2			+
CDS-ATP8	192	7786	7977	1	ATG	TAA	+
CDS-ATP6	681	7947	8627	−31	ATG	TAA	+
CDS-COIII	785	8627	9411	−1	ATG	TA—	+
tRNA-Gly	67	9411	9477	−1			+
CDS-ND3	348	9479	9826	1	ATC	TAA	+
tRNA-Arg	70	9828	9897	1			+
CDS-ND4L	297	9900	10,196	2	ATG	TAA	+
CDS-ND4	1375	10,190	11,564	−7	ATG	T——	+
tRNA-His	70	11,565	11,634	0			+
tRNA-Ser	59	11,635	11,693	0			+
tRNA-Leu	70	11,694	11,763	0			+
CDS-ND5	1824	11,755	13,578	−9	ATA	TAA	+
CDS-ND6	528	14,102	13,575	−4	ATA	TAG	−
tRNA-Glu	69	14,174	14,106	3			−
CDS-CytB	1140	14,178	15,317	3	ATG	TAG	+
tRNA-Thr	69	15,318	15,386	0			+
tRNA-Pro	67	15,455	15,389	2			−
Control Region	908	15,456	16,363	0			+

+ and − represent heavy and light strand, respectively.

### Phylogenetic analysis

We analyzed five newly sequenced mitochondrial genomes of the Laotian Rock Rat together with mitogenomes of six species, which are most closely related to the species according to Huchon et al. (2007), downloaded from GenBank. Thirteen protein-coding sequences and two rRNAs (12S and 16S) were individually aligned with MAFFT v7.158b (Katoh & Standley 2013), maintaining the open reading frames of the protein coding genes. The individual alignments were concatenated and divided into four partitions, including the rRNA region and 1st, 2nd, and, 3rd codon positions of the protein-coding genes.

A Bayesian analysis of the concatenated partitions (rRNA region and 1st, 2nd, and, 3rd codon positions of the protein coding genes) was performed with MrBayes v3.2.6 (Ronquist et al. 2012). A GTR model (Lanave et al. 1984) with gamma-distributed rate variation across sites Yang (1994) and a proportion on invariable sites was used. The posterior distributions of the parameters were estimated using Markov chain Monte Carlo (MCMC) sampling with three heated and one cold chain. Two independent MCMC runs were performed with 10 million generations per run and trees and parameters were sampled every 1000 generations. The first 25% samples were discarded as burn-in. Convergence to the stationary distribution was confirmed with Tracer v1.6 after examining the frequency plots and the effective sample size values.

To perform the maximum likelihood phylogenetic analysis, RAxML v8.0.26 (Stamatakis 2014) was used. The GTR + GAMMA model of nucleotide substitution was selected. Each one of the four data partitions (rRNA region and 1st, 2nd, and, 3rd codon positions of the protein-coding genes) was allowed to have a unique substitution rate. Bootstrap analysis with 100 pseudo-replicates was used to evaluate the support for the different nodes in the final tree.

### Time divergence estimation

To estimate divergence times between the clades, BEAST2 v2.3.2 (Bouckaert et al. 2014) was used. The split between the Ctenodactylidae and Diatomyidae (*Laonastes*) estimated at ∼44.3 ± 3.5 MYA was used as the calibration point (Huchon et al. 2007). A relaxed clock model (Drummond et al. 2006) was selected to take into account possible variation rates among branches, and Yule and Coalescent Constant Population Size (Heled & Drummond 2011) were set as the tree priors. Independent substitution models were assumed for each one of the four partitions. The posterior distribution parameters were estimated using MCMC sampling. Four independent runs of 100 million generations each were carried out; trees were sampled every 1000 generations. The first 10% generations were discarded as burn-in after inspection of trace plots and ESS scores with Tracer v1.6. The trees and log files were combined using LogCombiner v2.3.2 (included in the BEAST2 package). A maximum clade credibility chronogram tree was generated with TreeAnnotator v1.4.7 (included in the BEAST2 package) and visualized with FigTree v1.3.1.

## Results and discussion

The topologies inferred from Bayesian and maximum likelihood analyses were identical and robustly supported (Figure 1). Our phylogenetic results between the analyzed species are also consistent with those from Huchon et al. (2007). The divergence time between Laos and Vietnam’s population is estimated to be 7.9 MYA (95% confidence interval from 6.1 to 9.8) under the Yule process prior setting and 8.0 MYA (95% confidence interval from 6.2 to 10.2) under the Coalescent Constant Size prior setting. The estimates are similar to the calibrations by Nicolas et al. (2012), but younger than from those from Le et al. (2015) probably because the taxonomic and data sampling were limited in the latter study.

The results of this study confirm a long temporal isolation of the two major lineages within the Laotian Rock Rat. Such a deep divergence is surprising, as the Laotian Rock Rat itself is endemic to the Annamite Mountain range and only has a limited distribution. Previous studies suggested that the restriction of this species to karst habitats and the repeated erosion of their limestone habitat during the Mio-, Plio and Pleistocene resulted in natural population fragmentation (Nicolas et al. 2012; Le et al. 2015). The ancient Miocene divergence supports earlier suggestions of a taxonomic revision of *Laonastes*. The pairwise divergence of approximately 13.6–13.8% between the two lineages, estimated across the entire mitogenomes (Supplementary material), is similar if not greater than estimates between other mammalian congeneric species (Johns & Avise 1998). Further nuclear DNA sequencing and morphological data are needed, but if such data supports our findings the Vietnamese/Eastern Laos Rock Rat should be formally described as a sister species to *Laonastes aenigmamus*.

Our data highlight the conservation importance of the Annamite Range. Globally, no other regions had more large mammal species discovered during the last decades, e.g. Saola (Vu et al. 1993) and large-antlered Muntjac (Do et al. 1994). The deep split between the populations within the Annamite range further signifies that additional surveys in the field and molecular studies are needed to understand the complex evolutionary history of species in the Annamites. Particularly, little surveyed limestone formations in the Annamites need further conservation attention, as it is likely that additional surveys will result in new discoveries of species and populations.
